# Supplementary material for: Acute Respiratory Distress Syndrome Definitions in Adults and Children: A Comparative Narrative Review
Source: J Clin Med. 2025 Oct 28;14(21):7644. doi: 10.3390/jcm14217644 (PMC12607997; doi:10.3390/jcm14217644)
Supplement: Supplementary file 1 [file jcm-14-07644-s001.zip › jcm-3906642-supplementary.pdf]

Supplemental Material S1:

Full search strings (platform-adapted queries)

Search strings were adapted to each database's controlled vocabulary and field syntax.

1. MEDLINE/PubMed — General ARDS definitions

("acute respiratory distress syndrome"[MeSH Terms] OR ARDS OR "acute lung injury") AND (definition OR criteria OR "case definition" OR "diagnostic criteria") AND (adult OR pediatric OR children OR PALICC OR Berlin OR AECC OR Kigali OR "oxygenation index" OR OI OR OSI OR "SpO2/FiO2")
Filters: Humans; English/Spanish; 1967/01/01–2025/08/31

2. Embase — General ARDS definitions

('acute respiratory distress syndrome'/exp OR ards:ab,ti OR 'acute lung injury':ab,ti) AND (definition:ab,ti OR criteria:ab,ti OR 'case definition':ab,ti OR 'diagnostic criteria':ab,ti) AND (adult:ab,ti OR pediatric:ab,ti OR children:ab,ti OR palicc:ab,ti OR berlin:ab,ti OR aecc:ab,ti OR kigali:ab,ti OR 'oxygenation index':ab,ti OR oi:ab,ti OR osi:ab,ti OR 'spo2/fio2':ab,ti)
Limits: Humans; English/Spanish; 1967–2025

3. Web of Science Core Collection — General ARDS definitions

TS=("acute respiratory distress syndrome" OR ARDS OR "acute lung injury") AND TS=(definition OR criteria OR "case definition" OR "diagnostic criteria") AND TS=(adult OR pediatric OR children OR PALICC OR Berlin OR AECC OR Kigali OR "oxygenation index" OR OI OR OSI OR "SpO2/FiO2")
Timespan: 1967–2025; Language: English/Spanish

4. Scopus — General ARDS definitions

TITLE-ABS-KEY("acute respiratory distress syndrome" OR ARDS OR "acute lung injury") AND TITLE-ABS-KEY(definition OR criteria OR "case definition" OR "diagnostic criteria") AND TITLE-ABS-KEY(adult OR pediatric OR children OR PALICC OR Berlin OR AECC OR Kigali OR "oxygenation index" OR OI OR OSI OR "SpO2/FiO2")
Years: 1967–2025; Language: English/Spanish

5. Cochrane Library — General ARDS definitions

("acute respiratory distress syndrome" OR ARDS OR "acute lung injury") in Title Abstract Keyword AND (definition OR criteria OR "case definition" OR "diagnostic criteria") in Title Abstract Keyword AND (adult OR pediatric OR children OR PALICC OR Berlin OR AECC OR Kigali OR "oxygenation index" OR OI OR OSI OR "SpO2/FiO2") in Title Abstract Keyword
Date range: 1967–2025; Language: English/Spanish

Gender/Sex in ARDS — Diagnostic and prognostic queries

The following queries focus on sex/gender differences in diagnosis and prognosis of ARDS. Neonatal respiratory distress syndrome is excluded.

6. MEDLINE/PubMed — Diagnosis

((\"Acute Respiratory Distress Syndrome"[MeSH] OR ARDS OR "pediatric acute respiratory distress syndrome" OR PARDS OR PALICC) AND ("Sex Factors"[MeSH] OR "Sex Characteristics"[MeSH] OR "Gender Identity"[MeSH] OR sex OR gender OR "sex-based" OR "gender-based" OR male OR female OR men OR women) AND ("Diagnosis"[MeSH] OR diagnosis OR "diagnostic criteria" OR "case definition" OR "Diagnostic Errors"[MeSH] OR misclassification OR "Sensitivity and Specificity"[MeSH] OR "PaO2/FiO2" OR "SpO2/FiO2" OR OI OR OSI OR HFNO OR "high-flow nasal" OR "lung ultrasound" OR "chest radiography")) NOT "Respiratory Distress Syndrome, Newborn"[MeSH]
Filters: Humans; English/Spanish; 1967/01/01–2025/08/31

7. MEDLINE/PubMed — Prognosis

(("Acute Respiratory Distress Syndrome"[MeSH] OR ARDS OR PARDS OR PALICC) AND ("Sex Factors"[MeSH] OR "Sex Characteristics"[MeSH] OR "Gender Identity"[MeSH] OR sex OR gender OR "sex-based" OR "gender-based" OR male OR female OR men OR women) AND (prognosis OR "Prognosis"[MeSH] OR outcome* OR mortality OR "Hospital Mortality"[MeSH] OR survival OR "Length of Stay"[MeSH] OR "Ventilator-Free Days" OR severity OR "Disease Severity Index"[MeSH])) NOT "Respiratory Distress Syndrome, Newborn"[MeSH]
Filters: Humans; English/Spanish; 1967/01/01–2025/08/31

8. Embase — Diagnosis

('acute respiratory distress syndrome'/exp OR ards:ti,ab OR 'pediatric acute respiratory distress syndrome'/exp OR pards:ti,ab OR palicc:ti,ab) AND ('sex difference'/exp OR 'sex factor'/exp OR 'gender identity'/exp OR sex:ti,ab OR gender:ti,ab OR 'sex-based':ti,ab OR 'gender-based':ti,ab OR male:ti,ab OR female:ti,ab) AND ('diagnosis'/exp OR diagnosis:ti,ab OR 'diagnostic accuracy'/exp OR 'diagnostic error'/exp OR misclassification:ti,ab OR 'sensitivity and specificity'/exp OR 'paO2/fiO2':ti,ab OR 'spo2/fiO2':ti,ab OR oi:ti,ab OR osi:ti,ab OR hfno:ti,ab OR 'lung ultrasound':ti,ab OR 'chest radiography':ti,ab) NOT ('respiratory distress syndrome, newborn'/exp) AND [humans]/lim AND ([english]/lim OR [spanish]/lim) AND [1967-2025]/py

9. Embase — Prognosis

('acute respiratory distress syndrome'/exp OR ards:ti,ab OR 'pediatric acute respiratory distress syndrome'/exp OR pards:ti,ab) AND ('sex difference'/exp OR 'sex factor'/exp OR 'gender identity'/exp OR sex:ti,ab OR gender:ti,ab OR 'sex-based':ti,ab OR 'gender-based':ti,ab OR male:ti,ab OR female:ti,ab) AND (prognosis/exp OR mortality/exp OR survival/exp OR 'length of stay'/exp OR 'ventilator weaning'/exp OR outcome*:ti,ab OR severity:ti,ab) NOT ('respiratory distress syndrome, newborn'/exp) AND [humans]/lim AND ([english]/lim OR [spanish]/lim) AND [1967-2025]/py

10. Web of Science — Diagnosis

TS=("acute respiratory distress syndrome" OR ARDS OR "pediatric acute respiratory distress syndrome" OR PARDS OR PALICC) AND TS=(sex OR gender OR "sex-based" OR "gender-based" OR male OR female OR men OR women) AND TS=(diagnosis OR "diagnostic criteria" OR "case definition" OR misclassification OR "sensitivity and specificity" OR "PaO2/FiO2" OR "SpO2/FiO2" OR OI OR OSI OR HFNO OR "lung ultrasound" OR "chest radiography") NOT TS=("respiratory distress syndrome, newborn")
Timespan: 1967–2025; Languages: English OR Spanish; Document Types: Article/Review

11.Web of Science — Prognosis

TS=("acute respiratory distress syndrome" OR ARDS OR PARDS OR PALICC) AND TS=(sex OR gender OR "sex-based" OR "gender-based" OR male OR female OR men OR women) AND TS=(prognosis OR outcome* OR mortality OR survival OR "length of stay" OR "ventilator-free days" OR severity) NOT TS=("respiratory distress syndrome, newborn")
Timespan: 1967–2025; Languages: English OR Spanish

12.Scopus — Diagnosis

TITLE-ABS-KEY("acute respiratory distress syndrome" OR ARDS OR "pediatric acute respiratory distress syndrome" OR PARDS OR PALICC) AND TITLE-ABS-KEY(sex OR gender OR "sex-based" OR "gender-based" OR male OR female OR men OR women) AND TITLE-ABS-KEY(diagnosis OR "diagnostic criteria" OR "case definition" OR misclassification OR "sensitivity and specificity" OR "PaO2/FiO2" OR "SpO2/FiO2" OR OI OR OSI OR HFNO OR "lung ultrasound" OR "chest radiography") AND NOT TITLE-ABS-KEY("respiratory distress syndrome, newborn") AND (LIMIT-TO(LANGUAGE, "English") OR LIMIT-TO(LANGUAGE, "Spanish")) AND PUBYEAR > 1966 AND PUBYEAR < 2026

13.Scopus — Prognosis

TITLE-ABS-KEY("acute respiratory distress syndrome" OR ARDS OR PARDS OR PALICC) AND TITLE-ABS-KEY(sex OR gender OR "sex-based" OR "gender-based" OR male OR female OR men OR women) AND TITLE-ABS-KEY(prognosis OR outcome* OR mortality OR survival OR "length of stay" OR "ventilator-free days" OR severity) AND NOT TITLE-ABS-KEY("respiratory distress syndrome, newborn") AND (LIMIT-TO(LANGUAGE, "English") OR LIMIT-TO(LANGUAGE, "Spanish")) AND PUBYEAR > 1966 AND PUBYEAR < 2026

14.Cochrane Library — Diagnosis

("acute respiratory distress syndrome" OR ARDS OR "pediatric acute respiratory distress syndrome" OR PARDS OR PALICC):ti,ab,kw AND (sex OR gender OR "sex-based" OR "gender-based" OR male OR female OR men OR women):ti,ab,kw AND (diagnosis OR "diagnostic criteria" OR "case definition" OR misclassification OR "sensitivity and specificity" OR "PaO2/FiO2" OR "SpO2/FiO2" OR OI OR OSI OR HFNO OR "lung ultrasound" OR "chest radiography"):ti,ab,kw NOT ("respiratory distress syndrome, newborn"):ti,ab,kw

15.Cochrane Library — Prognosis

("acute respiratory distress syndrome" OR ARDS OR PARDS OR PALICC):ti,ab,kw AND (sex OR gender OR "sex-based" OR "gender-based" OR male OR female OR men OR women):ti,ab,kw AND (prognosis OR outcome* OR mortality OR survival OR "length of stay" OR "ventilator-free days" OR severity):ti,ab,kw NOT ("respiratory distress syndrome, newborn"):ti,ab,kw
